# Evaluation of CAN Bus Security Challenges †

**DOI:** 10.3390/s20082364

**Published:** 2020-04-21

**Authors:** Mehmet Bozdal, Mohammad Samie, Sohaib Aslam, Ian Jennions

**Affiliations:** IVHM Centre, Cranfield University, Cranfield MK43 0AL, UK; m.samie@cranfield.ac.uk (M.S.); S.Aslam@cranfield.ac.uk (S.A.); i.jennions@cranfield.ac.uk (I.J.)

**Keywords:** CAN network, CAN security, ECU, in-vehicle communication

## Abstract

The automobile industry no longer relies on pure mechanical systems; instead, it benefits from many smart features based on advanced embedded electronics. Although the rise in electronics and connectivity has improved comfort, functionality, and safe driving, it has also created new attack surfaces to penetrate the in-vehicle communication network, which was initially designed as a close loop system. For such applications, the Controller Area Network (CAN) is the most-widely used communication protocol, which still suffers from various security issues because of the lack of encryption and authentication. As a result, any malicious/hijacked node can cause catastrophic accidents and financial loss. This paper analyses the CAN bus comprehensively to provide an outlook on security concerns. It also presents the security vulnerabilities of the CAN and a state-of-the-art attack surface with cases of implemented attack scenarios and goes through different solutions that assist in attack prevention, mainly based on an intrusion detection system (IDS).

## 1. Introduction

The vehicle industry has evolved drastically over the last couple of decades into extensive automation of cars with a mesh of sensors and computational systems. These sensors are controlled by embedded electronic control units (ECUs), designed for the optimal management of a wide array of functions ranging from engine control to Anti-lock Braking (ABS) and Advanced Driver-Assistance Systems (ADAS), respectively. According to [1,2], a modern automobile is fitted with more than a hundred ECUs, and this number is envisaged to increase in the future. These ECUs are distributed all around the vehicle and communicate with each other via in-vehicle communication networks such as a Controller Area Network (CAN). Being the most common in-vehicle communication protocol for vehicle applications, CAN offers advantages such as cost-effective wiring, immunity to electrical interference, self-diagnosing, and error correction.

However, despite these functional benefits, the rising inter- and intra-vehicle communications render CAN vulnerable to cyber-attacks. The existing built-in security features of the CAN bus are primarily designed for ensuring reliable communication, and not for cybersecurity; therefore, it cannot prevent the network from cyberattacks. As a result, far-reaching implications of cyberattacks on CAN are anticipated. For instance, the safety of the driver and passengers can be jeopardised by the attack on airbag [3] or ABS systems. Eventually, it may affect the reputation of the car manufacturer with substantial financial implications like recalls [4]. Tampering of ECUs (e.g., used-cars’ odometers [5]) is yet another example that may result in dire consequences for consumers and manufacturers.

Equally alarming is the lack of encryption in CAN, which has a strong bearing on individual data privacy. By design, CAN is a broadcast network that allows nodes to capture messages going through the network. As the broadcasted data is not encrypted, an adversary can acquire the desired data. This may lead to an invasion of privacy, mainly when modern cars are capable of acquiring the driver’s personal information.

According to the 2019 industry survey [6], safety and security are the highest short-term and mid-term challenges for the automotive industry. Therefore, extensive studies have been carried out to find possible solutions [7,8] to the vulnerabilities of CAN. Some of these studies have performed successful experimental attacks on passenger cars [9,10,11,12,13,14] and heavy-duty vehicles [15,16]. At the same time, researchers have also proposed preventative methods for such known attacks. These include network segmentation, encryption, authentication, and intrusion detection systems (IDSs).

In light of the above, this paper provides a comprehensive literature review with the following main contributions:

- Identification of the state-of-the-art and the most-probable security challenges associated with modern vehicles, covering a number of implemented physical and remote access attacks.
- Highlighting the attack surfaces of modern vehicles with a critique on possible future attacks.
- An in-depth analysis of the current research on CAN security issues to facilitate their effective and optimal mitigation.

Accordingly, the rest of the paper is organised as follows. Section 2 provides a background study on the CAN, followed by Section 3, which presents a detailed vulnerability assessment of the CAN. In Section 4, we give an in-depth account of the attacks that have been implemented on the CAN network, followed by a critique of the existing and proposed solutions in Section 5. Finally, the research challenges are discussed in Section 6, with our conclusions given in Section 7.

## 2. Overview of the Controller Area Network (CAN)

The CAN bus is a multi-master broadcast communication protocol developed by Robert Bosch GmbH in the early 1980s. A traditional CAN interface can provide up to 1 Mbps [17]. In 2012, Bosch released the CAN FD (flexible data-rate), which can achieve 5 Mbps in practice and has a 64-byte payload compared to 8 bytes in the classical CAN [18]. CAN FD is backward compatible and can coexist with classical CAN nodes. Classical CAN and CAN FD are both standardised under ISO 11898-1:2015.

**Figure 1:**
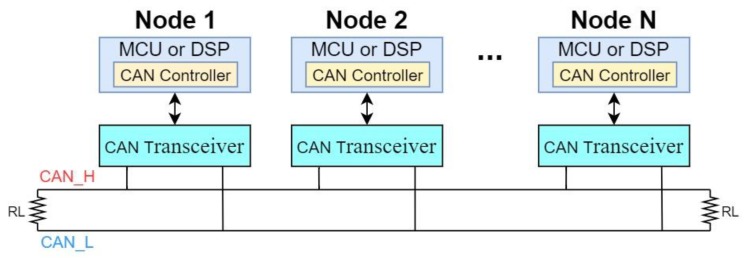
An example of a single two-wire Controller Area Network (CAN).

The single two-wire bus architecture of CAN, as shown in Figure 1, reduces cabling. The distributed architecture of the network provides easy-maintenance and decreases the overall system cost. Moreover, the protocol uses differential wiring mode, represented by CAN_H and CAN_L, which enhances the immunity to noise and electrical interference. From a logic point of view, signals have two states (voltage levels): A dominant logic ‘0’ and a recessive logic ‘1’, meaning that the bus signal remains ‘0’, the dominant logic, as long as one of the nodes releases logic ‘0’ to the bus.

**Figure 2:**
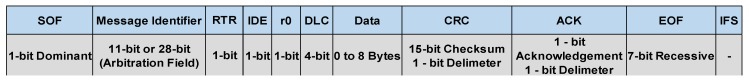
Classical CAN frame structure.

The CAN protocol has message-based communication provided via frames, as shown in Figure 2. Each frame has a message identifier field, data field, cyclic redundancy checksum (CRC), and some control bits. Every node listens to each frame and processes the relevant ones based on the message identifier field, which is also used for the arbitration.

### Reliable Communication in CAN

**Figure 3:**
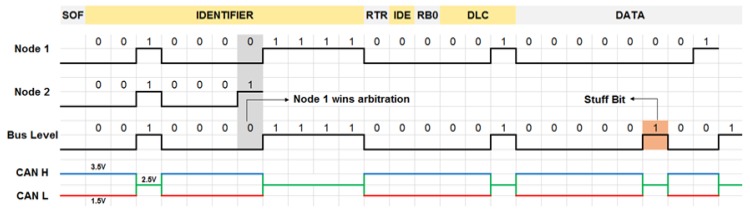
Signalling in CAN; Node 1 wins arbitration without any disruption.

The CAN protocol has a set of built-in features that provide robust communication. If two nodes start transmitting at the same time, the non-destructive arbitration mechanism resolves the conflict by allowing the highest priority node to continue the transmission without any interruption (e.g., Node 1 wins arbitration in Figure 3, without any disruption, as the dominant bit overrides the recessive one). Another feature is carrier sense multiple access with collision avoidance (CSMA/CA), which rules that the nodes have to wait for a certain amount of inactivity before the transmission. This assists in sensing if the bus is idle for ensuring that a collision will not occur.

The CAN bus has some bit-level and message-level error checking mechanisms. In the bit-level, the transmitter node monitors the bus. An error arises if there is a difference between the transmitted bit and the one observed on the bus. On the other hand, the message-level CAN-bus error check mechanism includes frame check over acknowledgment (ACK), cyclic redundancy checksum (CRC), and end of frame (EOF) fields. After the transmission of a frame, the transmitter node writes a recessive bit to the ACK field. If a node receives a message correctly, it overwrites the ACK field with a dominant bit; otherwise, the ACK field stays recessive, which indicates a transmission error. There is up to a 21-bit CRC field in a CAN frame for data integrity. If any node calculates a different CRC than the transmitter node, an error flag will be sent. The CRC delimiter, ACK delimiter, and EOF bits have fixed values and must always be recessive. During the frame form check, if these bits are dominant, an error is generated.

**Figure 4:**
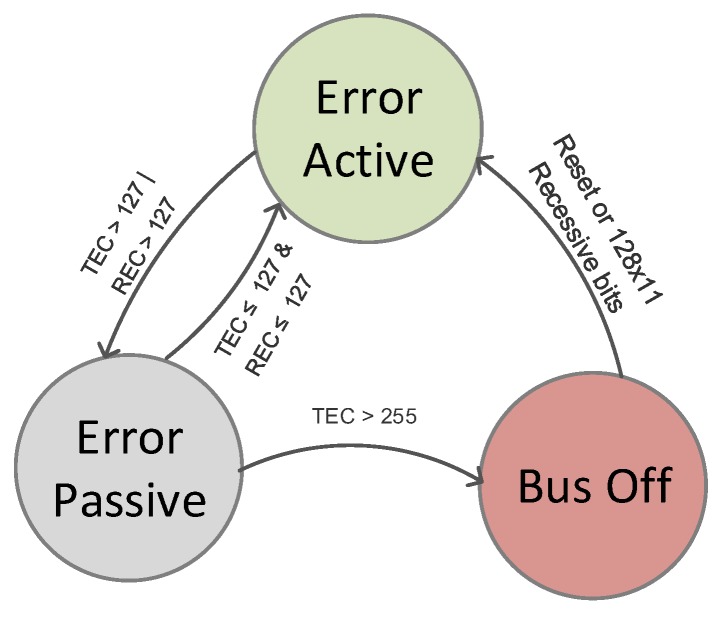
The state diagram of the error confinement mechanism (ECM) in the CAN bus.

CAN also prevents the physical errors by disabling the faulty nodes from the bus traffic with an error confinement mechanism (ECM), as shown in Figure 4. The ECM is facilitated in each node using two error counters known as the received error counter (REC) and transmitted error counter (TEC). The TEC increases by eight if an error occurs during the transmission, and REC increases by one if the error comes during the reception. Every successful transmission or reception of a frame decreases the responsible counter by one. The counters’ default values are zero, and nodes start at the error active state. A node will enter the error passive state if the value of the node’s counter exceeds 127. In the error passive state, the node can only write recessive error flags, which will not affect the bus traffic. The node turns to the bus off state if the TEC counter exceeds 255, meaning that the affected node will no longer take part in the bus traffic.

## 3. Vulnerability Assessment of the CAN Protocol

It is essential to have a vulnerability assessment of a network to highlight security problems. Therefore, the vulnerability assessment of the CAN protocol can be carried out based on confidentiality, integrity, and availability.

Confidentiality means providing the data only to authorised people. However, the CAN protocol does not have inherent cryptographic methods to ensure confidentiality. This allows an intruder to access sensitive user data and cause an invasion of privacy.

Integrity is the accuracy, completeness, and validity of the data. The CAN bus has a CRC for verification of integrity against the transmission errors, but it cannot prevent data injected by malicious parties, which breaks the integrity. The protocol does not have a comprehensive integrity check and fails to sustain integrity.

Availability means that authorised users can use the system at all times. Given the nature of priority-based messaging, if a message with the highest priority is transmitted/inserted, the network will be inaccessible by the lower priority nodes, and availability is violated.

The CAN bus failed to pass all three essential security criteria. Thus, it is a clear indication that the CAN protocol does not have any security measurements against the attacks.

## 4. Automotive Attack Surface and Existent Attacks

**Figure 5:**
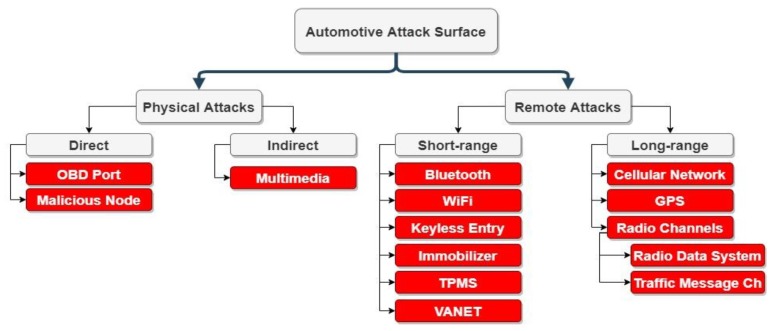
The automotive attack surface.

In the 1950s, automotive electronics cost only 1% of the total car cost, while it is currently 35% and is expected to rise to 50% in 2030 [19]. Although the rise in electronics has improved comfort, functionality, and driving safety, it has created new attack surfaces, as shown in Figure 5. The protocol itself is defenceless to attacks; therefore, any exploit in the current/future telematics unit or infotainment system can disrupt the network, as summarised in Table 1.

The first CAN bus attack was performed on the power window by Hoppe and Dittman in 2007 [7,25]. Since then, numerous attacks have been performed. These attacks can be categorised as physical access attacks, where the attacker should access the vehicle physically, or remote attacks, which are implemented via wireless communication interfaces. Although attacks in the literature are mainly physical access ones, some experts have argued that physical access to the CAN network is not practical [26]. Therefore, current research is mainly focusing on remote access attacks.

### 4.1. Physical Access Attacks

Physical access attacks require direct or indirect access to the CAN bus network. Direct access can be obtained by the On-Board Diagnostic (OBD) port or a malicious node. The OBD port is the primary attack surface; hence, it has access to all of the nodes, even though network segmentation is used.

Koscher et al. [10] manipulated the CAN and controlled various modules including essential brake control and engine control modules through the On-Board Diagnostics II (OBD-II) port. They released the brake and prevented its activation while the car was running 40 mph by the continuous fuzzing method. The attack also includes the manipulation of the instrument cluster with false data, changing engine parameters, and disabling the engine.

Due to the CAN architecture, any malicious node can listen or send a message to disrupt the network. The attacks implemented through the OBD port can be replicated using a malicious node. Palanca et al. [11] applied a selective denial-of-service (DoS) attack on an unmodified 2012 Alfa Romeo Giulietta. The research showed that any person who has physical access to the network can disrupt it, even with a simple tool. This attack does not require a full message transmission; instead, it overwrites to the recessive bits and generates a transmission error. The contribution of this research is that it exploited the vulnerability of the CAN standard. After this research, an alert (ICS-ALERT-17-209-01) [27] was announced by the U.S. government. A similar research analysis was carried out by Murvay and Groza [28] to show the limitations of the attack on different bit rates and to breach the authentication methods.

Mukherjee et al. [16] implemented DoS attacks on the SAE J1939 standard [29], which is used in heavy-duty commercial vehicles. They performed three separate DoS attacks: (i) sending too many request messages for a supported Parameter Group Number (PGN) to overload the recipient ECU, (ii) sending manipulated false request to send (RTS) and causing overflow at the recipient buffer, and (iii) keeping the connections open via Clear to Send (CTS) messages and occupying the whole network. This work was one of the first studies to exploit the SAE J1939 specification. Murvay and Groza [15] implemented impersonation and DoS attacks on SAE J1939. These works showed that SAE J1939 is vulnerable to protocol-specific attacks in addition to all CAN bus attacks.

There can also be indirect physical access attacks. These attacks require a physical object to be inserted into the car, but adversaries do not necessarily have direct access to the network. Checkoway et al. [20] developed an indirect access attack model, which included hacking the IT system of the car service and accessing the CAN via computer. The attack model also included attacking via multimedia devices (CD, USB, or MP3 player). Hoppe et al. [12] implemented an attack with a multimedia disc. Although the attack did not breach the CAN, it may scare the driver by flashing a warning on the screen and playing an alarm signal.

### 4.2. Remote Access Attacks

Nowadays, modern vehicles contain different types of wireless interfaces needed for communicating with systems such as passive anti-theft, tire pressure monitoring system (TPMS), Bluetooth, radio data, telematics, and so on. These wireless interfaces need to communicate with the CAN, usually via a gateway ECU to protect the network. However, there are studies that have demonstrated the hacking of a gateway ECU and gain accessed to the isolated CAN [12].

Checkoway et al. [20] compromised the TPMS, Bluetooth, FM channel, and a cellular network of a car through reverse engineering and claimed that thieves could steal vehicles easily as doors could be unlocked through the CAN messages. Woo et al. [21] proposed a remote attack via a malicious self-diagnostic app. If someone uses a malicious app to monitor/diagnose the vehicle’s situation, the adversary takes control of the vehicle remotely and performs its attack from a long-distance.

Valasek and Miller [22] carried out a remote attack survey on 12 car brands and 21 commercial cars and identified the remote attack surfaces and their difficulties in compromising each vehicle. The attack was three-staged. The first stage was to compromise the ECU responsible for a wireless interface. The second stage was to inject messages to communicate with the safety-critical ECU. The last stage was to modify the ECU to behave maliciously. While the researchers believed that the increasing number of cyber-physical systems in the cars would increase their vulnerabilities, they could not practically verify this because of the high number of different applications in the vehicles. Furthermore, they also hacked a Jeep Cherokee remotely and disabled the engine in 2014 [9]. After this attack, a public announcement that stated the vulnerability of motor vehicles against remote attacks was published [30].

Savage and his team [31] took control of a Chevrolet Corvette’s brakes and windshield wipers via a commercial telematics control unit in 2016. This attack indicates that the vulnerability of the CAN can be penetrated by the aftermarket equipment and cannot be entirely addressed by the manufacturer [32].

Nie et al. [13] implemented a remote attack on a Tesla Model S in 2016 via a wireless and cellular interfaces. The Keen Security Lab of Tencent [14] discovered multiple attack surfaces on BMW vehicles, which showed that even high-end commercially available cars could suffer from cyber-attacks.

Another wireless attack method is over-the-air (OTA) software updates. OTA is a cost-effective and scalable solution that allows the manufacturers to deliver software updates remotely. However, it is another attack surface where hackers can dive into the vehicle’s communication network. Beek and Samani [23] implemented a ransomware attack via an OTA update.

The remote attack surface of the modern car is more substantial than the physical one, and with the rising connectivity in cars, the number of wireless attack surfaces is increasing day by day. In the near future, cars will be equipped with vehicle-to-vehicle (V2V) and vehicle-to-infrastructure (V2I) communications, which build vehicular ad hoc networks (VANETs). VANETs aim for traffic optimisation and collision avoidance. To provide these benefits, VANETs use car sensors and have wireless connectivity. In VANETs, spoofed messages can be received or transmitted, and as a result, the in-vehicle communication network may be disrupted.

### 4.3. Privacy in the CAN

Acquiring CAN network data not only causes safety issues, but also the invasion of privacy. The modern vehicle collects data related to the driver, which passes through the vulnerable CAN network. An investigation [33] revealed that it was possible to obtain the precise location history of the car and other personal data (log of phone calls, list of contacts, email addresses, and photos) from the connected phone. An adversary can steal personal information only by passively listening to the bus. Furthermore, researchers [34,35] have shown that it is possible to identify the driver based on the sensory data travelling through the CAN bus. Therefore, monitoring the in-vehicle network can invade personal privacy.

## 5. Counter Measures for CAN Attacks

The attacks on CAN clearly show that the protocol is very vulnerable and requires cyber defence mechanisms for safe driving. The studies to solve this problem have mainly focused on four defence mechanisms: network segmentation, encryption, authentication, and intrusion detection, which are summarised in Table 2.

### 5.1. Network Segmentation

The most straightforward protection mechanism is separating the CAN network into multiple subnetworks. The segmentation provides control over who can access particular subnetwork and reduce the damage of the attack by limiting its spread. The interconnection between subnetworks is controlled via a gateway ECU. This model currently exists in commercial vehicles. The method is simple to implement, but it is not effective if the gateway ECU is compromised or manipulated like the hacking exhibited in [12]. Kammerer et al. [36] addressed this issue and proposed a star coupling router with security features. The paper ignored the security inside a subnetwork, but it is possible to implement a replay attack in a subnetwork and attack the other subnetworks bypassing the security check of the router.

Researchers at TU München proposed an automotive service bus architecture [37] where their two-layer architecture was designed to prevent external attacks. The infotainment system and all vital functions were separated from each other. All components could send and receive messages, but by default, they could not send any data as the central ECU allows whom to write to the automotive service bus.

Network segmentation increases the security level, but it is not a sufficient method to protect the CAN. It also makes the maintenance of the system more difficult, along with the increased cost.

### 5.2. Encryption

The CAN protocol uses a shared broadcast network without a built-in encryption mechanism. This allows an adversary to eavesdrop all the nodes and understand the communication. To prevent this data breach, a light-weight encryption system should be implemented. Although there are commercial software-based encryption methods (e.g., Trillium [38], CANcrypt [39]) and manufacturers have proprietary encryption techniques implemented in cars, there have been reports claiming that encryption mechanisms in commercially available cars can be broken [40,41].

The limited data field is one of the problems for secure CAN encryption. This problem can be overcome by sending multiple CAN frames for a single message, and may solve the problem on low traffic networks, but it is not a solution for the currently rising traffic in automobile CAN networks. Another issue is the limited computational power of ECUs. If we consider the lifetime of a vehicle, it is possible to crack a static encryption key. Therefore, dynamic key exchange is required. However, this is harder to implement and is computationally expensive. The dynamic key can also cause latency on resource-constrained ECUs, and it is not acceptable for safety-critical real-time systems.

The different encryption mechanisms proposed are shown in Table 3. Doan and Ganesan [42] implemented hardware-based AES-128 encryption on FPGA chips for the CAN system. The hardware implementation of the method decreases latency and increases throughput. However, the method changes the legacy ECU and is not backward compatible. Another study used physical unclonable functions (PUFs) [43]. This method can obtain the private key from the physical characteristics of the ECUs; thus, hiding the key is not a problem. Although the method solves the problem for generating encryption keys, it also requires modifying the ECU.

Encryption hardens attacks and provides privacy; however, it is not sufficient to protect the CAN. Even the unbreakable encryption mechanism cannot prevent replay attacks.

### 5.3. Authentication

It is not possible to identify the sender of a CAN message. If an adversary has access to the network, they can send malicious messages and all the nodes accept them as authentic. This can be prevented via authentication.

VeCure [48] authentication, which has an acceptable 50 us processing delay, is based on trust groups where high-trust groups share a symmetric secret key. The method has a major advantage with fewer key numbers, which corresponds in size to the number of trust groups rather than the ECU number. However, it sends an authentication message after every transmitted frame, which doubles the network traffic. Another drawback of the method is that it cannot protect the system if a node from the trust group is compromised. LiBrA-CAN [49], proposed by Groza et al., splits the authentication keys between groups of multiple nodes to improve efficiency. Although the method is quite successful, it requires high bandwidth and is not compatible with traditional CAN.

Nowdehi et al. [50] identified five criteria for an authentication method to be implemented commercially: cost-effectiveness, backward compatibility, support for vehicle repair and maintenance, sufficient implementation details, and acceptable overhead. They analysed ten authentication methods in the literature using them. Not surprisingly, none of the methods could pass all five criteria.

There are also off-the-shelf products providing hardware-based authentication like the S32K family from NXP [51]. The S32K family has Cryptographic Service Engine (CSE), which has a Cipher-based Message Authentication Code (CMAC) to provide secure authentication, and is a hardware-based system that accelerates the process drastically. For instance, public-key authentication can be achieved in less than 100 us [52] with hardware acceleration, while software authentication takes more than 10 ms, depending on the key size. However, the industry is currently concerned with the cost of ECUs. With the enhancement of hardware technology, it is possible to see more hardware-based methods to secure the CAN.

### 5.4. Intrusion Detection System (IDS)

Implementing security features on a safety-critical real-time system is a difficult task. Strong cryptographic methods are not feasible due to the limited resources (memory, bandwidth, and computational power) and time constraints, from which research on intrusion detection system (IDS) for CAN has emerged. The main advantage of IDS is that it usually does not modify the current CAN controller and the bus traffic does not increase.

Intrusion detection methods can be categorised as signature-based (misuse) detection and anomaly-based detection [53]. Signature-based detection checks for known attacks on the database; therefore, it requires regular updates for new attacks. Although it is quite successful in detecting known attacks, it fails to detect unknown attacks. Anomaly-based IDS analyses the behaviour of the network and recognises the deviation from normal behaviour. Accuracy is usually lower than that of the signature-based. In contrast to signature-based detection, anomaly-based IDS may detect unknown attacks.

There are different parameters that an IDS system can assess on the CAN. Müter et al. [54] defined eight anomaly detection sensors, as shown in Table 4, to identify the anomalies in a structured way. All these detection sensors were inspired by the typical behaviour of the CAN bus. Deviation from the normal behaviour of these sensors is the sign of the intrusion, and different IDS solutions use these sensors to detect intrusions. These solutions can be categorised as time/frequency-based, physical system characteristic, specification-based, and feature-based.

#### 5.4.1. Time/Frequency-Based IDS

The automobiles have rigid safety rules and most of the ECUs transmit periodic signals. Any change in the frequency can be interpreted as abnormal behaviour, in other words, an intrusion. The basic IDS analyses the frequency of the CAN messages as presented in [55,56].

Offset ratio and time interval based IDS [57], as proposed by Lee et al., analyses the response time of the transmitted remote frame where the simple effective algorithm can detect attacks and type of attacks, however, the method increases bus traffic by injecting remote frames for analyses.

The time/frequency analysis provides useful information about the CAN. However, the vehicle’s situation (e.g., idle, running) and the priority scheme of the CAN may significantly change the timing information and affect the result of time/frequency-based IDS. The method also cannot detect attacks where the frequency is not changed, like a masquerade attack in [58].

#### 5.4.2. Physical Characteristic Based IDS

The physical characteristic of the CAN network can be used to detect intrusions; hence each transceiver has a different signal shape even though they transmit the same data. This can be caused by random manufacturing variations, cabling, and aging.

In [59], Choi et al. proposed VoltageIDS, which uses unique electrical characteristics of the CAN signal like a fingerprint. The different locations of the ECUs with different lengths of wire results in different resistance [60] and the resistance changes the signal features. They analysed eight of the signal features like positive and negative slope values and voltage value at a dominant level. The method has zero false-positive rates and can differentiate between attacks and errors; however, it requires an oscilloscope to gather the network signal and has heavy signal processing.

The CAN does not have a shared master clock, and each ECU uses its own quartz crystal. Cho and Shin [58] suggested the use of clock skew to detect intrusions. Although ECUs run the same frequency, they may have random drifting exceeding 2400 ms in a day [61]. They fingerprinted the transmitter ECU via the clock skew and detected the intrusions. Although they could reach 97% of the anomaly detection with a false-positive rate of 0.55%, the method only worked for the periodic messages. However, this method can be tricked by mimicking the clock skew, as shown in [62].

The physical characteristics of the CAN provides substantial information about ECUs. However, environmental factors like temperature and humidity and aging of the components can change the physical characteristics; therefore, the IDS may fail. They can also not detect the attacks from the software layer because the authenticated ECU will transmit the malicious messages, and the IDS does not find any changes to the signal characteristics. Similarly, the physical characteristic-based IDS requires heavy signal processing. As a result, it may cause latency or require expensive hardware.

#### 5.4.3. Specification-Based IDS

Larson et al. [63] suggested specification based attack detection and implemented specification rules based on the CAN Open protocol. This method has limited attack detection capability and requires all the ECUs to have detectors. The method also is not powerful enough to prevent attacks; hence there are protocol compliant attacks like in [64].

Studnia et al. [65] proposed a language-based intrusion detection and derived the language characteristic of the network from the ECUs’ specifications and generated the forbidden sequences. If one of these sequences occurs, an intrusion is detected.

#### 5.4.4. Feature-Based IDS

Feature-based system analysis examines the network parameters like busload, frequency, number of dropped messages, and other parameters like abnormal messages and payload. This is usually based on artificial intelligence techniques.

Generative adversarial nets (GAN) based IDS [66] was proposed by Seo et al., who used the deep-learning model. The method is easy to expand and difficult to manipulate by an attacker, hence the detection mechanism has a black-box characteristic. Bloom filtering [67], proposed by Groza and Murvay, analysed the periodicity and payload of CAN messages. This method provides a memory-efficient analysis of data. Although both methods require heavy computation, they look promising in terms of tackling the CAN security problem.

Table 5 presents the comparison of the IDSs. Each method has a unique feature to suppress other methods, but also comes with a cost. For example, physical characteristic-based IDS can easily detect an inauthentic node, but it fails to detect an attack from a software layer. The best IDS system should be a hybrid system that takes advantage of different methods. Although IDS can mitigate a security problem, it cannot provide confidentiality. To have complete security, cryptography is required.

## 6. Discussions on CAN Security Research

Automotive security is getting more attention, and standardisations are coming to tackle cybersecurity problems. Cybersecurity guidebook for cyber-physical vehicle systems [73] and the fundamental principles of automotive cybersecurity specification (PAS 1885:2018) [74] were published by SAE in 2016 and British Standards Institute in 2018 consecutively. ISO 21434 Automotive Cybersecurity [75] is under development and expected to be released by 2020.

The CAN protocol has also gained attention from the industry to its vulnerabilities, and companies are now manufacturing high-end secure ECUs. The Secure Hardware Extension (SHE) [76] specification developed by the Hersteller Initiative (HIS) becomes an open standard and used by many companies in their ECUs like NXP MPC5646C [52] microcontroller. Some commercial ECUs have built-in IDS; the NXP TJA115x [77] series can prevent spoofing attacks and be used as an IDS. There are also commercial proprietary intrusion detection systems [78,79].

Although there have been steps taken to protect the CAN, there is still more to do. The industry does not share some of their research, and academia does not have enough resources. As such, there are not sufficient attack data and benchmarks. Implementing attacks on real vehicles can be unfeasible for safety concerns and cost. To overcome these challenges, there should be more research on modelling CAN bus attacks like in [80] and creating attack databases like in [81,82]. Sharing datasets as an open-source (e.g., like in [66]) will help researchers; hence working on shared datasets will give a reference point to compare their research.

## 7. Conclusions

The CAN protocol facilitating ECUs in modern vehicles is not geared up and well-protected against the complex and evolving nature of cyberattacks. The existing security features incorporated in vehicles are not fit and adequate to resist and defy them. This is attributable to the lack of encryption and authentication mechanisms, which provide multiple opportunities for several types of attacks to materialize and as a result, jeopardize the individual data privacy and the safety of the vehicle occupants. These blemish the manufacturers’ reputation and downgrade vehicle reliability, followed by substantial financial losses.

We have observed that the existing trend of attacks is mainly physical-access oriented; however, with the growing connectivity in vehicles, we have also noted a considerable increase in wireless attacks. This developing trend is indicative of wireless attacks outpacing the physical access attacks in the near future.

Moreover, an in-depth analysis of the vulnerabilities of the CAN bus to cyberattacks points to the limitations posed by the protocol. The root cause evaluation of various attacks and the critique of potential solutions has revealed the inadequacies and constraints of both the industry and academia. They are not driven toward mutual sharing of an attack database, allocation of testing and trial resources, and developing benchmarks for an open-source.

There are four main countermeasures for the CAN attacks, comprising network segmentation, encryption, authentication, and IDS. They are, however, heavy on overheads with respect to the availability of the existing resources. Further analysis has revealed IDS as the most promising option when compared to the rest of the solutions above-mentioned. It is noteworthy that the IDS may not provide complete security, but it can prevent several of the CAN vulnerabilities with acceptable overhead. We presume that future vehicles will have IDS solutions not only to secure the vehicle, but to also provide data to the manufacturer to tackle cyberattacks.

## Figures and Tables

**Table 1:** Summary of the Controlled Area Network (CAN) bus attacks.

Reference	DoS	Modification ^1^	Access Type	Notes/Root Cause
[11]	Y	N	OBD II	Does not require full CAN messages
[20]	N	Y	OBD II, CD, Bluetooth, GSM	Systematical experimental attacks. Indirect access via the car service computer
[21]	N	Y	In-direct OBD II	Attack via a smartphone app
[22]	Y	Y	Multiple remote sources	Remote attack analysis of 21 commercial cars
[13]	N	Y	Wi-Fi, GSM	Access CAN network via a browser exploit
[16]	Y	N	OBD II, compromised ECU	SAE J1939 data-link layer exploits
[23]	N	Y	Wi-Fi, GSM	Ransomware attack over the air
[24]	N	Y	TPMS	Remotely sending false TPMS data

^1^ The modification includes replay, impersonation, and bogus information attacks.

**Table 2:** Methods to secure the CAN bus.

Proposed Method	Benefits	Disadvantages
Network Segmentation	Limit access to the end-user	Increased cost, Difficulty in maintenance
Encryption	Hardened attacks, Confidential data transmission	Increased computational power, Increased traffic, Weak encryption due to frame size
Authentication	Secure data transmission	Increased computational power, Increased traffic
Intrusion Detection	Detect anomalies and attacks	Complicated algorithm design, Cannot guarantee the security

**Table 3:** Encryption methods for the CAN bus.

Reference	Encryption Method	Traffic Effect	Key
[42]	AES-128 and SHA-1	Increased	Static Symmetric
[44]	XOR	No Change	Dynamically Synchronised
[43]	AES-256 and Elliptic-curve Diffie–Hellman	Increased	Symmetric
[45]	XOR	No Change	Static Symmetric
[46]	Tiny Encryption Algorithm	Increased	Static Symmetric
[47]	Triple DES	Increased	Dynamically Synchronised

**Table 4:** Automotive anomaly detection sensors [54].

Sensor	Description
Formality	Correct message size, header and field size, field delimiters, checksum, etc.
Location	The message is allowed with respect to the dedicated bus system
Range	Compliance of payload in terms of data range
Frequency	Timing behaviour of messages is approved
Correlation	Correlation of messages on different bus systems adheres to the specification
Protocol	The correct order, start-time, etc. of internal challenge-response protocols
Plausibility	Content of message payload is plausible, no infeasible correlation with previous values
Consistency	Data from redundant sources is consistent

**Table 5:** Comparison of the intrusion detection system (IDS).

Reference	Algorithm Analyses	Parameters	Advantages	Downsides
[66]	Generative Adversarial Nets	A pattern of CAN ID	CAN train itself for unknown attacks	Expensive hardware
[68]	Adaptive Network-based Fuzzy Inference System	Busload, message frequency analysis	Detect attack type, simple solution	Works for simple attacks, updated each second, needs a feature database
[69]	Entropy-based	Entropy of IDs, payload	Does not require much information about traffic data	Very vulnerable to some attacks which include random bits
[70]	Long Short-term Memory Networks	Payload	Does not require pre-knowledge	Does not understand the natural change
[63]	Specification-based	Protocol policy	Less dependency	IDS should be placed at every ECU
[71]	Hamming Distance	Payload	Low computation	Low detection
[57]	Offset ratio and time interval	Remote frame timing	Simple efficient algorithm with low-cost hardware	Increased traffic
[72]	Analysis of ID Sequence	Sequence of ID	Low memory and computation requirement, detection of inserted few malicious messages	Very vulnerable to attacks which have a similar sequence of normal traffic
[59]	Support Vector Machine and Boosted Decision Tree	Electrical signal	Robust to some attack types, first IDS to differentiate between an error and an attack	High cost and vulnerable to environmental changes
[58]	Recursive Least Squares	Clock skew	Robust to some attack types,	Only works on periodic signals
[67]	Bloom Filtering	Message identifier, payload	Low memory usage for membership testing	Complex algorithm
[56]	Probability Density Function	Reception cycle period ( frequency analysis)	Online learning	Hard to authenticate a non-periodic message
[55]	Flow-based	Message frequency	Simple algorithm	Only works on periodic signals
